# An Integrated Multi-Biomarker Perspective on Acute Exercise: Differential Orexin, Alpha-Amylase, and Cortisol Responses Across Modalities

**DOI:** 10.3390/sports14080351

**Published:** 2026-08-13

**Authors:** Fiorenzo Moscatelli, Vincenzo Monda, Marco La Marra, Antonietta Messina, Antonietta Monda, Maria Casillo, Nicola Mancini, Girolamo Di Maio, Gabriella Marsala, Giovanni Messina, Rita Polito

**Affiliations:** 1 Department of Education and Sport Sciences, Pegaso Telematic University, 80143 Naples, Italy; fiorenzo.moscatelli@unipegaso.it (F.M.); nicola.mancini@unipegaso.it (N.M.); 2 Department of Economics, Law, Cybersecurity, and Sports Sciences, University of Naples “Parthenope”, 80131 Naples, Italy; vincenzo.monda@uniparthenope.it; 3 Section of Human Physiology, Unit of Dietetics and Sports Medicine, Department of Experimental Medicine, University of Campania “Luigi Vanvitelli”, 80138 Naples, Italy; marco.lamarra@unicampania.it (M.L.M.); maria.casillo@unicampania.it (M.C.); 4 Department of Precision Medicine, University of Campania “Luigi Vanvitelli”, 80138 Naples, Italy; antonietta.messina@unicampania.it; 5 Department of Human Science and Quality of Life Promotion, San Raffaele Telematic University, 00166 Rome, Italy; antonietta.monda@uniroma5.it; 6 Department of Psychology and Health Sciences, Pegaso Telematic University, 80143 Naples, Italyrita.polito@unipegaso.it (R.P.); 7 Dipartimento del Farmaco Asp di Catania, 95131 Catania, Italy; gabriella.marsala@aspct.it

**Keywords:** orexin-A, salivary alpha-amylase, cortisol, high-intensity interval training, exercise physiology, neuroendocrine response

## Abstract

**Purpose:** Acute exercise elicits coordinated neuroendocrine and autonomic responses that are strongly influenced by exercise modality and intensity. While cortisol and salivary alpha-amylase are widely used as peripheral indicators of hypothalamic–pituitary–adrenal (HPA) axis and sympathetic nervous system activation, respectively, salivary orexin remains an exploratory biomarker whose relationship with central orexinergic activity is not yet fully established, particularly in response to different exercise conditions. This study aimed to compare the acute salivary responses of orexin, alpha-amylase, and cortisol following distinct exercise modalities characterized by different physiological loads. **Methods:** A repeated-measures design was employed in a sample of physically active young women. Each participant completed four experimental sessions: high-intensity interval training (HIIT), combined exercise (CE), moderate-intensity continuous training (MICT), and yoga. Saliva samples were collected at baseline (T0), immediately post-exercise (T1), and during early recovery (T2 and T3). Biomarker concentrations were analyzed to assess temporal dynamics and modality-dependent differences. **Results:** All biomarkers showed modality-dependent responses. HIIT elicited the most pronounced increases in alpha-amylase and cortisol, reflecting robust sympathetic and HPA axis activation, whereas CE and MICT induced more moderate responses. Yoga was associated with minimal changes across all biomarkers. Orexin displayed a graded response across the exercise modalities, with higher post-exercise levels observed following HIIT compared to lower-intensity conditions. Temporal analyses revealed distinct kinetic profiles, with alpha-amylase peaking rapidly in the early post-exercise phase, whereas cortisol exhibited a more delayed response. **Conclusions:** These findings demonstrate that acute salivary responses of orexin, alpha-amylase, and cortisol are strongly modulated by exercise modality, supporting the importance of an integrated, multi-biomarker approach to characterize the physiological response to exercise. The inclusion of salivary orexin provides exploratory information on a potentially relevant arousal- and energy-regulation-related pathway, complementing established peripheral markers of autonomic and endocrine activation. This integrated perspective may contribute to a more comprehensive understanding of exercise-induced stress responses in young women.

## 1. Introduction

Physical exercise is a potent physiological stimulus capable of modulating neuroendocrine, autonomic, and stress-related pathways. The magnitude and temporal profile of these responses depend on several factors, including exercise intensity, duration, modality, and the characteristics of the recovery period. In recent years, increasing attention has been directed toward the use of salivary biomarkers as practical and non-invasive tools for monitoring these acute responses, both in laboratory settings and in applied sport and exercise contexts. Saliva offers a valuable alternative to blood sampling for the repeated assessment of several endocrine and stress-related markers, including cortisol and alpha-amylase, and is particularly suitable when multiple post-exercise time points are required [1]. At the same time, current evidence indicates that salivary responses are not uniform, but vary substantially according to the physiological load imposed by exercise and to the kinetics of recovery.

Among salivary biomarkers, cortisol remains one of the most widely used indicators of hypothalamic–pituitary–adrenal (HPA) axis activation. As a glucocorticoid released in response to physical and psychological challenges, cortisol plays a central role in energy mobilization, metabolic regulation, and adaptation to stress. Exercise is a well-recognized activator of the HPA axis, yet the direction and magnitude of the cortisol response depend strongly on exercise intensity and context [2]. Evidence suggests that more vigorous exercise elicits a more pronounced endocrine response than lighter workloads, supporting the idea that exercise intensity is a key determinant of acute stress-related hormonal dynamics. Because cortisol also follows a marked circadian rhythm, rigorous standardization of sampling conditions is essential when interpreting exercise-induced changes.

Alongside cortisol, salivary alpha-amylase has emerged as a useful marker of sympathetic and adrenergic activation [1]. Unlike cortisol, which primarily reflects the HPA axis, alpha-amylase is considered more closely related to the immediate autonomic component of the stress response. Acute exercise, especially when strenuous or intermittent, has been associated with rapid increases in salivary alpha-amylase, consistent with heightened sympathetic nervous system activity. This makes alpha-amylase particularly informative when the aim is to characterize the early post-exercise phase and to distinguish between exercise modalities that differ in cardiovascular and perceptual demand. Although the literature supports its responsiveness to acute physical stress, available evidence also indicates that its post-exercise profile is influenced by exercise characteristics and recovery timing, highlighting the importance of repeated measurements during the immediate recovery period [3,4,5].

A further, and considerably less explored, candidate biomarker in the context of exercise is orexin. Orexins, also known as hypocretins, are excitatory neuropeptides produced predominantly in the lateral hypothalamus and are involved in the regulation of wakefulness, arousal, energy expenditure, feeding behavior, autonomic function, and motivated behavior [5]. Their widespread central projections place the orexinergic system at the intersection of several functions that are relevant to exercise physiology, including vigilance, motor activation, cardiovascular regulation, and metabolic adaptation. However, salivary orexin represents a peripheral measurement and cannot be regarded as a direct index of hypothalamic orexinergic activity. Its use in exercise research should therefore be considered exploratory. Within this framework, concurrent assessment of salivary orexin and more established stress-related biomarkers may help generate hypotheses regarding the interaction between exercise load, arousal-related regulation, and peripheral neuroendocrine responses [4,6].

This rationale becomes even more compelling when considering that orexin appears to be functionally linked not only to physical activity and energy balance, but also to sympathetic regulation and cardiovascular control. Translational evidence suggests that the orexin system contributes to the integration of sleep–wake regulation, autonomic activation, and behavioral readiness and may therefore participate in the organism’s adaptive response to demanding physical stimuli. At the same time, the recent literature has emphasized that active lifestyles may influence orexin signaling, while orexin itself may contribute to the regulation of arousal, spontaneous activity, and metabolic engagement. Despite this growing conceptual framework, direct human evidence on acute exercise-induced changes in orexin remains limited, particularly in studies adopting controlled experimental designs and examining multiple exercise modalities within the same protocol [7,8,9].

An additional point of interest concerns the comparison between exercise modalities with clearly different physiological loads. High-intensity interval training (HIIT) is characterized by repeated vigorous efforts interspersed with recovery and is known to produce substantial metabolic, autonomic, and hormonal stress. Moderate-intensity continuous training (MICT), by contrast, imposes a more stable and less abrupt cardiovascular demand, while combined exercise integrates aerobic and resistance elements, potentially generating a mixed stimulus that engages both cardiovascular and neuromuscular systems. Yoga represents a markedly different condition, generally characterized by low-intensity movement, controlled breathing, and relaxation-oriented components. Importantly, evidence from systematic reviews indicates that even a single session of yoga or meditation-based practice may attenuate acute stress reactivity, supporting its role as a meaningful low-activation comparison condition rather than a simple passive control [10].

Despite the broad literature on exercise and stress physiology, an important gap remains. Most studies have primarily focused on isolated biomarkers, single exercise modalities, or longer-term training adaptations, whereas relatively few investigations have examined, within the same experimental framework, how distinct acute exercise conditions modulate a set of biomarkers reflecting complementary physiological domains. In particular, this has been achieved by integrating exploratory salivary orexin measurements with established peripheral markers of autonomic and endocrine activation. This approach may be especially informative in healthy, physically active young women, a population that remains relatively underrepresented in exercise biomarker research despite the well-recognized influence of sex-specific physiological factors on stress reactivity and recovery processes [11,12].

Therefore, the aim of the present study was to investigate and compare the acute salivary responses of orexin, alpha-amylase, and cortisol following exercise modalities characterized by distinct physiological loads, including high-intensity interval training, combined exercise, moderate-intensity continuous training, and yoga, in physically active young women. By examining both immediate and short-term post-exercise responses across multiple time points, this study sought to determine whether exercise modality and presumed physiological load differentially shape neuroendocrine and autonomic activation profiles. We hypothesized that the magnitude of the biomarker responses would follow a graded pattern according to overall physiological demand, with HIIT eliciting the most pronounced response, followed by combined exercise and MICT, while yoga would produce the smallest changes.

## 2. Materials and Methods

### 2.1. Participants

Fifteen healthy young women aged 20–30 years were recruited for this study. All participants were physically active in their daily lives but were not competitive athletes and were not engaged in structured training programs. This population was selected to investigate acute neuroendocrine, autonomic, and stress-related salivary responses in individuals accustomed to habitual physical activity but not specifically adapted to high-level athletic training loads. The sample size was determined by the exploratory nature of the study and the repeated-measures crossover design, in which each participant completed all four experimental conditions and therefore served as their own control. This design was expected to reduce the influence of between-subject variability and increase sensitivity to within-participant changes across exercise modalities and time points. Nevertheless, given the limited number of participants and the absence of a formal prospective power calculation, the findings should be regarded as preliminary and interpreted primarily on the basis of effect estimates and their confidence intervals. Before participation, all individuals were informed about the aims, procedures, and experimental requirements of the study and provided their consent to take part in all testing sessions. Descriptive characteristics of the sample are reported in Table 1. The study was conducted in accordance with the Declaration of Helsinki and was approved by the local Institutional Review Board (IRB) (Prot. 0013787/I, 23 April 2025). All participants provided written informed consent prior to participation.

### 2.2. Study Design

The study adopted a repeated-measures within-subject design. Each participant completed four different experimental exercise conditions (HIIT, CE, MICT, and yoga) in a randomized order: high-intensity interval training (HIIT), combined exercise (CE), moderate-intensity continuous training (MICT), and yoga. All participants performed every condition, and sessions were separated by a one-week washout period to minimize residual fatigue, carry-over effects, and possible interference between exercise modalities.

To reduce the influence of circadian fluctuations on salivary biomarkers, particularly cortisol, all experimental sessions were conducted in the morning between 9:00 a.m. and 12:00 p.m. Participants were instructed to maintain their usual lifestyle habits throughout the study period and to refrain from strenuous physical activity before each experimental session. Experimental conditions, scheduling, and sampling procedures were standardized across all sessions.

### 2.3. Exercise Protocols

The four exercise modalities were selected to provide clearly differentiated physiological stimuli, ranging from a low-intensity condition to progressively more demanding aerobic and interval-based exercise. All sessions were conducted under standardized laboratory conditions and were designed to elicit distinct acute physiological responses potentially associated with differences in salivary orexin, alpha-amylase, and cortisol concentrations.

Maximum heart rate (HRmax) was estimated using the age-predicted equation proposed by Tanaka et al. (208 − 0.7 × age), which has been shown to provide improved accuracy compared to traditional predictive formulas [13]. The session began with a 5 min warm-up performed at low intensity (~50–60% HRmax). Participants then completed 10 high-intensity intervals of 1 min each at ~85–95% HRmax, interspersed with 1 min of active recovery at ~50–60% HRmax. Exercise was performed on a motorized treadmill, with intensity continuously monitored using a chest-strap heart rate monitor. Participants were verbally encouraged to maintain the prescribed intensity.

The protocol resulted in a 1:1 work-to-recovery ratio, with a total session duration of approximately 25 min, including warm-up and recovery phases. This structure is consistent with established HIIT paradigms known to induce rapid autonomic and neuroendocrine activation [14].

The combined exercise (CE) session was structured as a standardized circuit integrating aerobic and resistance components. Following a 5 min warm-up at low intensity (~50–60% HRmax), participants performed a circuit consisting of five exercises: bodyweight squats, push-ups, alternating lunges, plank hold, and jumping jacks. Each exercise was performed for 40 s with 20 s of transition between exercises. The circuit was repeated three times, with a 2 min active recovery between circuits consisting of low-intensity walking. Exercise intensity was maintained at approximately 65–75% HRmax and continuously monitored using a chest-strap heart rate monitor. The total duration of the CE session was approximately 30 min, including warm-up and cool-down.

This standardized circuit protocol was designed to provide a combined aerobic and resistance stimulus while maintaining a moderate-to-vigorous exercise intensity throughout the session. The three-circuit structure, together with the prescribed work-to-rest ratio, was selected to elicit both cardiovascular and neuromuscular adaptations representative of combined exercise while ensuring consistency across participants [15]. The MICT session consisted of continuous aerobic exercise performed at moderate intensity. Exercise was conducted on a treadmill and began with a 5 min warm-up at low intensity (~50–60% HRmax).

Participants then performed continuous exercise for 30–40 min at ~65–75% HRmax, maintaining a steady workload throughout the session. Exercise intensity was monitored continuously using a chest-strap heart rate monitor (Polar Electro, Kempele, Finland) to ensure adherence to the target intensity zone. A 5 min cool-down at low intensity followed the main exercise phase, resulting in a total session duration of approximately 40–50 min. This protocol reflects standard moderate-intensity continuous training commonly used in experimental and clinical studies [16].

The yoga session consisted of a standardized and instructor-led sequence including breathing exercises (pranayama), controlled postural movements (asanas), and a final guided relaxation phase. The session began with 5 min of controlled breathing, followed by approximately 20 min of structured low-intensity postures focusing on mobility, balance, and controlled transitions. The final 5 min consisted of guided relaxation in a supine position. Participants were instructed to maintain slow, controlled breathing throughout the session. Exercise intensity remained below 50% HRmax and was continuously monitored. The session was conducted at low intensity and aimed to minimize sympathetic activation while promoting relaxation and parasympathetic engagement [17]. Heart rate was continuously monitored throughout each exercise session using a validated chest-strap heart-rate monitor. In addition to prescribing exercise intensity according to age-predicted maximal heart rate, internal physiological load was quantified by calculating mean heart rate, peak heart rate, mean and peak percentages of age-predicted maximal heart rate, time spent within the prescribed target heart-rate zone, and session rating of perceived exertion (Borg CR10 scale) recorded immediately after each exercise session.

Across all conditions, participants completed the assigned session according to the same weekly schedule and under comparable experimental circumstances. Although the expected physiological load differed substantially among modalities, all sessions were administered in a controlled and standardized manner.

### 2.4. Saliva Collection and Sampling Time Points

Salivary samples were collected to assess acute changes in orexin, alpha-amylase, and cortisol concentrations across the four exercise conditions. All collections were performed under standardized conditions in the morning (between 9:00 a.m. and 12:00 p.m.) to minimize circadian variability, particularly for cortisol. Participants were instructed to refrain from eating, consuming caffeine, smoking, and brushing their teeth for at least 2 h before each session. Water consumption was permitted before testing, provided that no fluids were consumed during the final 10 min preceding saliva collection, in accordance with current recommendations for standardized saliva sampling procedures [18]. Upon arrival at the laboratory, participants remained seated at rest for at least 10 min before baseline sampling. Saliva was collected using the passive drool method, which is considered the gold standard for salivary biomarker assessment, allowing uncontaminated whole saliva collection. Participants were instructed to allow saliva to accumulate in the mouth and then gently expel it into sterile polypropylene tubes.

Samples were collected at four time points for each experimental session:

• **T0**: Baseline, immediately before exercise;
• **T1**: Immediately post-exercise (within 3 min post-exercise);
• **T2**: 15 min post-exercise;
• **T3**: 30 min post-exercise.

This sampling schedule was selected to capture both the immediate response and the short-term recovery kinetics of salivary biomarkers, particularly cortisol and alpha-amylase, which are known to exhibit time-dependent post-exercise dynamics.

Immediately after collection, saliva samples were stored on ice and subsequently centrifuged at 3000 rpm for 10 min at 4 °C to remove particulate matter. The supernatant was aliquoted and stored at −80 °C until biochemical analysis. Salivary cortisol and alpha-amylase concentrations were determined using commercially available enzyme-linked immunosorbent assay (ELISA) kits (Salimetrics LLC, State College, PA, USA), following the manufacturer’s instructions. Orexin concentrations were measured using a specific ELISA kit (MyBioSource Inc., San Diego, CA, USA). The assay sensitivity was approximately 0.007 µg/dL, with intra- and inter-assay coefficients of variation below 5% and 10%, respectively. Salivary alpha-amylase activity was measured using a kinetic enzyme assay (e.g., Salimetrics LLC), based on the rate of substrate conversion, with results expressed in U/mL. The assay showed high sensitivity and reliability, with intra- and inter-assay coefficients of variation below 7%. Salivary orexin concentrations were quantified using a specific ELISA kit validated for salivary samples (e.g., MyBioSource or equivalent). The assay sensitivity was in the low pg/mL range, with intra- and inter-assay coefficients of variation below 10%. All samples were analyzed in duplicate, and the mean value was used for statistical analysis. To minimize inter-assay variability, all samples from the same participant were analyzed within the same assay batch. Laboratory analyses were performed by trained personnel blinded to the experimental conditions.

### 2.5. Outcome Measures

The primary outcomes were salivary concentrations of orexin, alpha-amylase, and cortisol measured at T0, T1, T2, and T3 under each of the four experimental conditions. These biomarkers were selected to investigate acute exercise-induced responses related to neuroendocrine activation, autonomic regulation, and stress-related endocrine dynamics.

### 2.6. Statistical Analysis

All statistical analyses were performed using Python 3.13 (64-bit), with the use of appropriate libraries for statistical modeling and data visualization (e.g., pandas, statsmodels, and matplotlib). Data were structured in long format, with each observation representing a unique combination of participant, exercise modality, biomarker, and time point. Data integrity was verified before analysis, confirming a fully balanced dataset with no missing observations, duplicate entries, or incomplete combinations.

For each biomarker, longitudinal changes were analysed using linear mixed-effects models. Participant was included as a grouping factor with a subject-specific random intercept, thereby accounting for the correlation among repeated observations obtained from the same individual. Fixed effects included Modality, Time, and the Modality × Time interaction. The random-effects variance–covariance structure consisted of a single random-intercept variance component, while the residual errors were assumed to be independent and to have constant variance conditional on the participant-specific random intercept and the fixed effects. No random slopes or additional within-subject covariance structures were included. Two coding approaches were used to characterize model effects. Sum coding was applied to evaluate omnibus effects of Modality, Time, and their interaction, whereas treatment coding was used to obtain interpretable contrasts, with yoga as the reference condition for Modality and T0 as the reference level for Time.

Model adequacy was assessed by verifying convergence and inspecting residual-versus-fitted plots, quantile–quantile plots of residuals, the distribution of subject-specific random effects, and the presence of influential or extreme observations. Residuals were examined for major deviations from normality, heteroscedasticity, and systematic temporal patterns. Post hoc comparisons were performed using estimated marginal means derived from the fitted models. Pairwise contrasts were computed between modalities at each time point and within each modality across time, with Holm adjustment applied to control the family-wise error rate. To verify that the four exercise protocols elicited distinct internal physiological loads, cardiovascular and perceptual variables (mean heart rate, peak heart rate, mean percentage of age-predicted maximal heart rate, peak percentage of age-predicted maximal heart rate, target-zone adherence, and session rating of perceived exertion [RPE]) were analysed using one-way repeated-measures analysis of variance (ANOVA), with exercise modality entered as the within-participant factor. Pairwise comparisons were adjusted using the Holm procedure. Effect size was quantified using partial eta squared (partial η^2^). Time spent within the prescribed target heart-rate zone was reported descriptively and was not subjected to inferential statistical analysis because the duration of the prescribed target period differed across exercise modalities.

To complement the longitudinal analyses, area under the curve (AUC) metrics were calculated for each participant and biomarker using the trapezoidal method based on actual time intervals. Time points were defined as T0 = baseline (pre-exercise), T1 = immediately post-exercise (0–3 min), T2 = 15 min post-exercise, and T3 = 30 min post-exercise. Two indices were derived: area under the curve with respect to ground (AUCg), representing total exposure, and area under the curve with respect to increase (AUCi), representing cumulative change relative to baseline. AUC values retained the units of the respective biomarker multiplied by time (e.g., pg·min/mL for orexin and cortisol, U·min/mL for alpha-amylase), thereby representing cumulative biomarker exposure or cumulative change over the observation period. AUC values were then analysed using linear mixed-effects models with Modality as a fixed effect and participant as a random intercept. Post hoc pairwise comparisons between modalities were again adjusted using the Holm procedure. Descriptive statistics are reported as means and standard deviations. Model results are presented as regression coefficients (β), standard errors (SEs), 95% confidence intervals (CIs), and *p*-values. Interpretation focused primarily on the magnitude and precision of the estimated effects, as reflected by regression coefficients, between-condition differences, and their 95% confidence intervals, rather than on statistical significance alone. *p*-values were considered complementary measures of statistical compatibility and were interpreted together with effect estimates and confidence intervals. Statistical significance was set at *p* < 0.05.

## 3. Results

### 3.1. Dataset Integrity and Preliminary Checks

The final dataset was fully balanced, comprising 720 observations from 15 participants, with no missing values, duplicated entries, or incomplete combinations across modality, parameter, and time. This ensured full suitability for mixed-effects modeling and longitudinal comparisons. Baseline (T0) values did not differ significantly across exercise modalities for any biomarker, confirming comparability of pre-exercise conditions. Additionally, no significant temporal variation was observed within the Yoga condition, supporting its role as a low-intensity control.

### 3.2. Exercise Intensity Verification

To verify that the four experimental protocols generated distinct physiological demands, heart-rate-derived variables and session ratings of perceived exertion were compared across exercise modalities (Table 2). Exercise modality significantly influenced all cardiovascular and perceptual indices of internal load (all *p* ≤ 0.019), confirming a clear physiological separation among the prescribed exercise conditions.

Mean heart rate across the entire exercise session was highest during HIIT (153.2 ± 7.4 bpm), followed by combined exercise (137.4 ± 6.8 bpm), MICT (132.6 ± 6.1 bpm), and yoga (87.9 ± 5.8 bpm), corresponding to 80.2 ± 3.8%, 71.9 ± 3.7%, 69.4 ± 3.3%, and 46.0 ± 3.1% of age-predicted maximal heart rate, respectively. Because HIIT incorporated active recovery periods, heart rate was also evaluated during the prescribed high-intensity intervals alone, yielding a mean intensity of 86.3 ± 3.5% of age-predicted maximal heart rate, confirming that participants consistently achieved the intended vigorous exercise stimulus.

Peak heart rate followed the same graded pattern, reaching 181.5 ± 5.2 bpm (95.0 ± 2.8% HRmax) during HIIT, 158.1 ± 7.2 bpm (82.8 ± 3.8% HRmax) during combined exercise, 147.6 ± 6.4 bpm (77.3 ± 3.4% HRmax) during MICT, and 102.6 ± 6.2 bpm (53.7 ± 3.3% HRmax) during yoga. Holm-adjusted post hoc comparisons confirmed that HIIT elicited greater cardiovascular demand than all other modalities, whereas combined exercise also produced significantly higher cardiovascular responses than MICT.

Analysis of adherence to the prescribed target heart-rate zones further confirmed the validity of the experimental protocols. During HIIT, participants spent 8.6 ± 0.9 min within the prescribed 85–95% HRmax interval, corresponding to 86.0 ± 9.0% adherence. During combined exercise and MICT, participants remained within the prescribed 65–75% HRmax zone for 20.9 ± 2.1 min and 32.8 ± 2.4 min, respectively, whereas yoga participants completed 26.4 ± 2.0 min below 50% HRmax. These findings demonstrated satisfactory adherence to the intended exercise intensity in all four protocols.

Session ratings of perceived exertion, assessed immediately after exercise using the Borg CR10 scale, closely paralleled the cardiovascular responses. Mean RPE values were 8.4 ± 0.8 following HIIT, 6.1 ± 0.9 following combined exercise, 4.3 ± 0.8 following MICT, and 2.0 ± 0.6 following yoga. Taken together, the cardiovascular and perceptual data confirmed that the four exercise protocols elicited clearly distinct internal physiological loads, supporting the interpretation that the observed salivary biomarker responses reflected genuine modality-dependent differences in exercise intensity rather than variability in protocol execution.

### 3.3. Orexin Response

The linear mixed-effects model revealed a significant Modality × Time interaction for salivary orexin, indicating that its temporal response differed across exercise conditions. Relative to yoga, HIIT produced an increase of 53.16 pg/mL at T1 (β = 53.16, SE = 5.47, 95% CI [42.43, 63.89], *p* < 0.001), 86.28 pg/mL at T2 (β = 86.28, SE = 5.47, 95% CI [75.55, 97.01], *p* < 0.001), and 104.95 pg/mL at T3 (β = 104.95, SE = 5.47, 95% CI [94.22, 115.68], *p* < 0.001). Combined exercise produced smaller but clearly estimated increases at T1 (β = 32.53, SE = 5.47, 95% CI [21.80, 43.26], *p* < 0.001), T2 (β = 56.03, SE = 5.47, 95% CI [45.30, 66.76], *p* < 0.001), and T3 (β = 70.69, SE = 5.47, 95% CI [59.96, 81.42], *p* < 0.001). The effects observed after MICT were more modest, amounting to 11.67 pg/mL at T1 (β = 11.67, SE = 5.47, 95% CI [0.94, 22.40], *p* = 0.033), 25.24 pg/mL at T2 (β = 25.24, SE = 5.47, 95% CI [14.51, 35.97], *p* < 0.001), and 37.43 pg/mL at T3 (β = 37.43, SE = 5.47, 95% CI [26.70, 48.16], *p* < 0.001). The magnitude and precision of these estimates indicated a progressively greater peripheral orexin response across MICT, combined exercise, and HIIT, whereas changes following yoga remained small.

No significant main effects of modality or time were detected in the absence of interaction, indicating that changes were driven by exercise-specific temporal dynamics.

**Figure 1:**
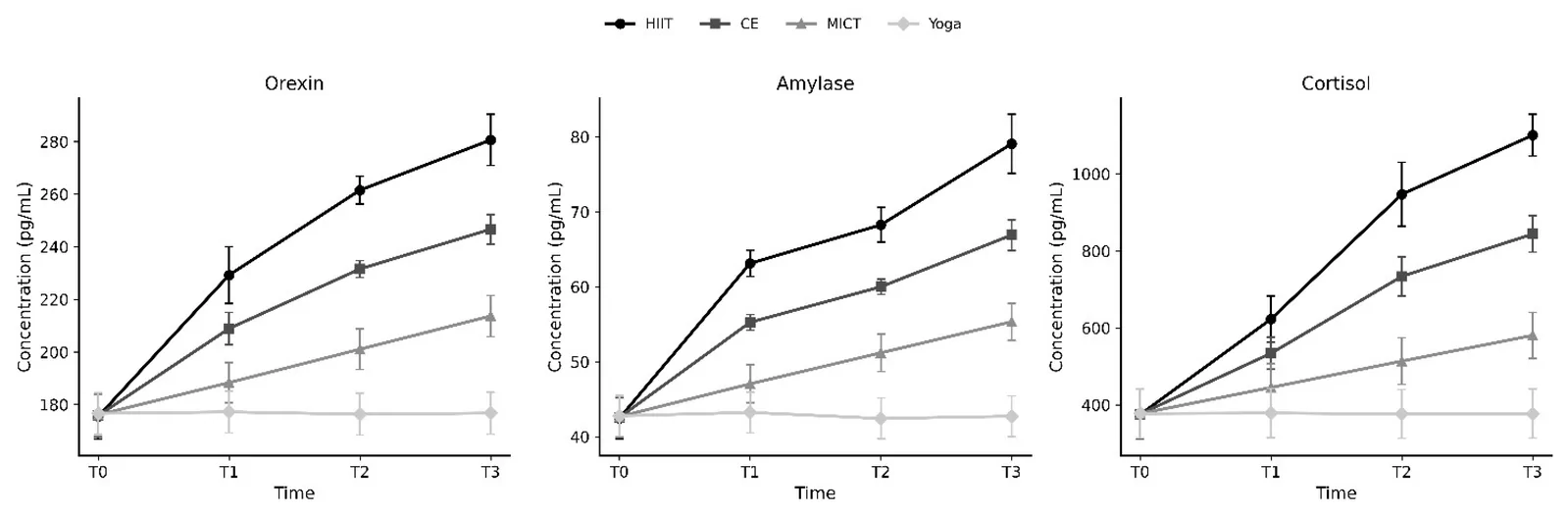
Time-course changes in salivary biomarkers following different exercise modalities. Mean concentrations of orexin, alpha-amylase, and cortisol (±95% confidence intervals) measured at baseline (T0) and at three post-exercise time points (T1, T2, T3) following four exercise conditions: high-intensity interval training (HIIT), combined exercise (CE), moderate-intensity continuous training (MICT), and yoga (control condition). Biomarker concentrations varied across exercise conditions and time points, with higher responses observed in HIIT compared with CE and MICT, whereas yoga showed minimal variation over time. Orexin and alpha-amylase increased progressively across post-exercise time points in the active conditions, whereas cortisol showed larger changes at T2 and T3. Values are expressed in the original measurement units for each biomarker (pg/mL for orexin and cortisol; U/mL for alpha-amylase). Data points represent group means, and error bars indicate 95% confidence intervals.

Holm-adjusted post hoc comparisons indicated no meaningful between-modality differences at baseline. From T1 onward, the estimated differences showed a consistent graded pattern, with the largest orexin response following HIIT, an intermediate response following CE, a smaller response following MICT, and minimal variation following yoga. The magnitude of the model-based estimates increased across the recovery period, particularly for HIIT and CE, and the corresponding confidence intervals remained clearly separated from the null value. Within-condition comparisons similarly indicated progressive increases from baseline after HIIT, CE, and MICT, whereas estimates for yoga remained small and imprecise around zero (Figure 1).

The AUC analysis supported the longitudinal findings. For AUCg, HIIT showed greater cumulative orexin exposure than combined exercise (Δ = 67.14 pg·min/mL, SE = 9.80, 95% CI [47.93, 86.35], Holm-adjusted *p* < 0.001), MICT (Δ = 134.55 pg·min/mL, SE = 9.80, 95% CI [115.34, 153.76], Holm-adjusted *p* < 0.001), and yoga (Δ = 188.65 pg·min/mL, SE = 9.80, 95% CI [169.44, 207.86], Holm-adjusted *p* < 0.001). A similar hierarchy emerged for AUCi, with HIIT exceeding combined exercise (Δ = 65.42 pg·min/mL, 95% CI [46.21, 84.63]), MICT (Δ = 130.86 pg·min/mL, 95% CI [111.65, 150.07]), and yoga (Δ = 183.74 pg·min/mL, 95% CI [164.53, 202.95]); all Holm-adjusted *p*-values were <0.001.

### 3.4. Alpha-Amylase Response

**Figure 2:**
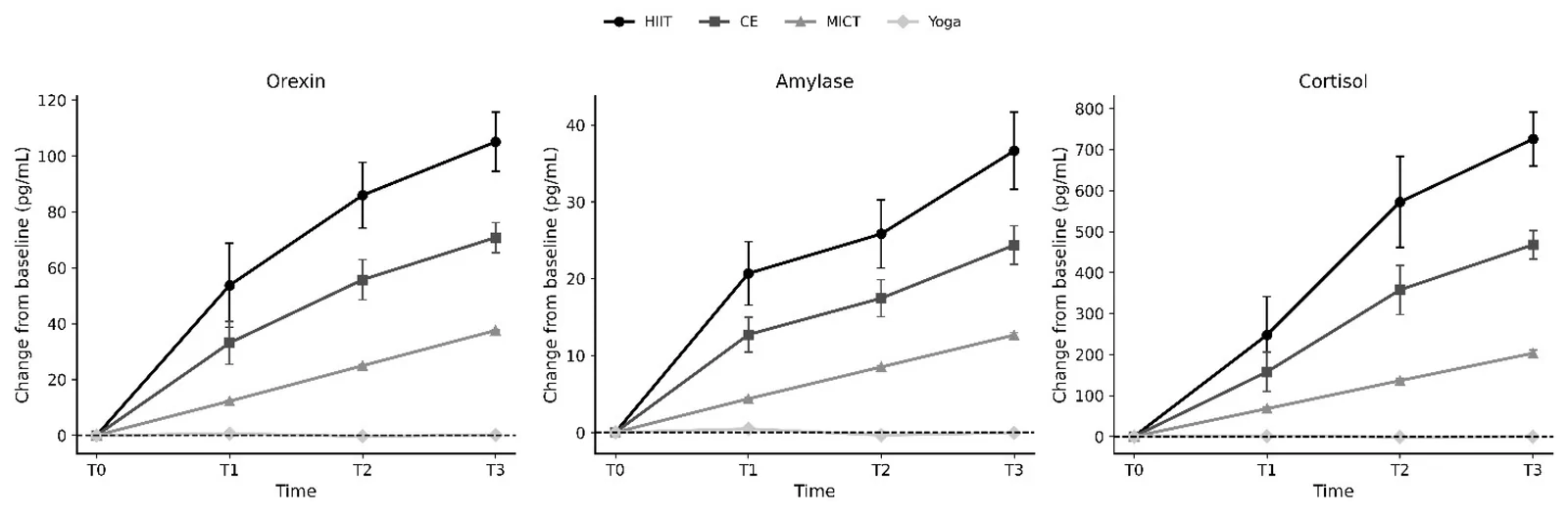
Changes from baseline in salivary biomarkers across exercise modalities. Mean changes from baseline (Δ; ±95% confidence intervals) in orexin, alpha-amylase, and cortisol concentrations measured at T1, T2, and T3 relative to pre-exercise values (T0), following four exercise conditions: high-intensity interval training (HIIT), combined exercise (CE), moderate-intensity continuous training (MICT), and yoga (control condition). All active exercise modalities showed increases from baseline, with larger changes observed in HIIT compared with CE and MICT, whereas yoga remained close to baseline values. Orexin and alpha-amylase exhibited progressive increases across post-exercise time points, while cortisol showed a more pronounced response at later time points (T2 and T3). The dashed horizontal line represents the baseline reference (Δ = 0). Values are expressed in the original measurement units for each biomarker (pg/mL for orexin and cortisol; U/mL for alpha-amylase). Data points represent group means, and error bars indicate 95% confidence intervals.

The linear mixed-effects model indicated a Modality × Time interaction for salivary alpha-amylase, showing that the magnitude of the post-exercise response differed across the four experimental conditions. Relative to yoga, HIIT produced an increase of 20.23 U/mL at T1 (β = 20.23, SE = 1.90, 95% CI [16.51, 23.95], *p* < 0.001), which increased to 34.68 U/mL at T2 (β = 34.68, SE = 1.90, 95% CI [30.96, 38.40], *p* < 0.001) and 43.91 U/mL at T3 (β = 43.91, SE = 1.90, 95% CI [40.19, 47.63], *p* < 0.001). Combined exercise elicited intermediate responses at T1 (β = 12.84, SE = 1.90, 95% CI [9.12, 16.56], *p* < 0.001), T2 (β = 22.67, SE = 1.90, 95% CI [18.95, 26.39], *p* < 0.001), and T3 (β = 29.36, SE = 1.90, 95% CI [25.64, 33.08], *p* < 0.001). MICT produced smaller but progressively increasing effects at T1 (β = 5.21, SE = 1.90, 95% CI [1.49, 8.93], *p* = 0.006), T2 (β = 11.43, SE = 1.90, 95% CI [7.71, 15.15], *p* < 0.001), and T3 (β = 16.72, SE = 1.90, 95% CI [13.00, 20.44], *p* < 0.001). Estimates for yoga remained close to zero across the observation period. Overall, the model-based estimates and their confidence intervals supported a graded modality-dependent response, with the greatest alpha-amylase activation following HIIT, intermediate activation following combined exercise, and a smaller response following MICT (Figure 2).

**Figure 3:**
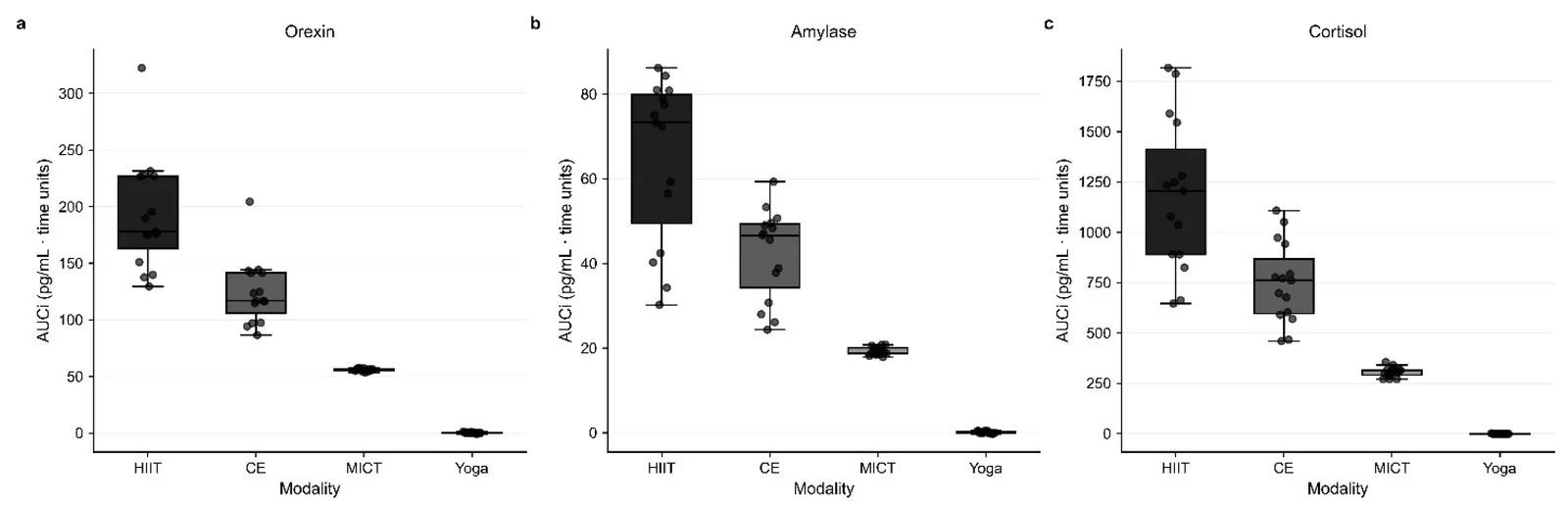
Cumulative biomarker responses across exercise modalities (AUCi). Area under the curve with respect to increase (AUCi) for orexin, alpha-amylase, and cortisol across the four exercise conditions: high-intensity interval training (HIIT), combined exercise (CE), moderate-intensity continuous training (MICT), and yoga (control condition). (**a**) orexin AUCi; (**b**) alpha-amylase AUCi; (**c**) cortisol AUCi. AUCi values represent the cumulative change in biomarker concentration relative to baseline (T0) across the post-exercise time points (T1–T3), providing an integrated measure of the overall physiological response. A clear modality-dependent gradient is observed across all biomarkers, with HIIT eliciting the highest cumulative responses, followed by CE and MICT, while yoga remains near baseline levels, indicating minimal activation. Box plots represent the median and interquartile range, with whiskers indicating the range of the data. Individual data points are overlaid to illustrate within-group variability. Values are expressed in biomarker-specific concentration-by-time units: pg·min/mL for orexin and cortisol and U·min/mL for alpha-amylase.

The AUC analyses supported the longitudinal results. HIIT showed greater AUCg than combined exercise (Δ = 22.18 U·min/mL, SE = 3.10, 95% CI [16.10, 28.26], Holm-adjusted *p* < 0.001), MICT (Δ = 44.94 U·min/mL, SE = 3.10, 95% CI [38.86, 51.02], Holm-adjusted *p* < 0.001), and yoga (Δ = 63.76 U·min/mL, SE = 3.10, 95% CI [57.68, 69.84], Holm-adjusted *p* < 0.001). The same hierarchy was observed for AUCi, with HIIT exceeding combined exercise (Δ = 21.36 U·min/mL, 95% CI [15.28, 27.44]), MICT (Δ = 43.17 U·min/mL, 95% CI [37.09, 49.25]), and yoga (Δ = 61.88 U·min/mL, 95% CI [55.80, 67.96]); all Holm-adjusted *p*-values were <0.001 (Figure 3).

### 3.5. Cortisol Response

Cortisol showed the largest absolute changes among the three biomarkers, with a clear Modality × Time interaction. Relative to yoga, HIIT produced an estimated increase of 246.88 pg/mL at T1 (β = 246.88, SE = 40.10, 95% CI [168.28, 325.48], *p* < 0.001). The magnitude of the difference increased markedly at T2 (β = 573.17, SE = 40.10, 95% CI [494.57, 651.77], *p* < 0.001) and T3 (β = 725.95, SE = 40.10, 95% CI [647.35, 804.55], *p* < 0.001). Combined exercise also elicited substantial, although smaller, increases at T1 (β = 168.42, SE = 40.10, 95% CI [89.82, 247.02], *p* < 0.001), T2 (β = 389.76, SE = 40.10, 95% CI [311.16, 468.36], *p* < 0.001), and T3 (β = 512.64, SE = 40.10, 95% CI [434.04, 591.24], *p* < 0.001). MICT produced a smaller immediate response at T1 (β = 67.00, SE = 40.10, 95% CI [−11.60, 145.60], *p* = 0.095), with the confidence interval including zero. Its response became more evident at T2 (β = 137.38, SE = 40.10, 95% CI [58.78, 215.98], *p* = 0.001) and T3 (β = 203.40, SE = 40.10, 95% CI [124.80, 282.00], *p* < 0.001). These estimates indicate that the cortisol response was both modality-dependent and temporally delayed, particularly following HIIT and combined exercise.

Baseline cortisol values were comparable across modalities. At T1, the estimated differences were already substantial for HIIT and CE relative to yoga, whereas the MICT estimate was smaller and its confidence interval was compatible with little or no immediate difference. At T2 and T3, the magnitude of the between-condition contrasts increased, producing a clear gradient across HIIT, CE, MICT, and yoga. Within-modality estimates showed progressive increases following HIIT and CE, while MICT displayed a delayed response that became more evident at T2 and T3. Yoga remained close to baseline throughout the observation period. The AUC analyses were consistent with the longitudinal findings. HIIT showed greater cortisol AUCg than combined exercise (Δ = 430.10 pg·min/mL, SE = 61.50, 95% CI [309.56, 550.64], Holm-adjusted *p* < 0.001), MICT (Δ = 870.24 pg·min/mL, SE = 61.50, 95% CI [749.70, 990.78], Holm-adjusted *p* < 0.001), and yoga (Δ = 1175.56 pg·min/mL, SE = 61.50, 95% CI [1055.02, 1296.10], Holm-adjusted *p* < 0.001). AUCi showed the same ordering, with HIIT exceeding combined exercise (Δ = 415.72 pg·min/mL, 95% CI [295.18, 536.26]), MICT (Δ = 842.61 pg·min/mL, 95% CI [722.07, 963.15]), and yoga (Δ = 1138.43 pg·min/mL, 95% CI [1017.89, 1258.97]); all principal Holm-adjusted comparisons yielded *p* < 0.001.

### 3.6. Overall Response Pattern

Across all biomarkers, a consistent pattern emerged. Baseline equivalence confirmed the absence of pre-intervention differences, while Yoga showed negligible variation over time. In contrast, the three active exercise modalities elicited significant, time-dependent increases, with a clear intensity gradient:

### 3.7. HIIT > CE > MICT > Yoga

**Figure 4:**
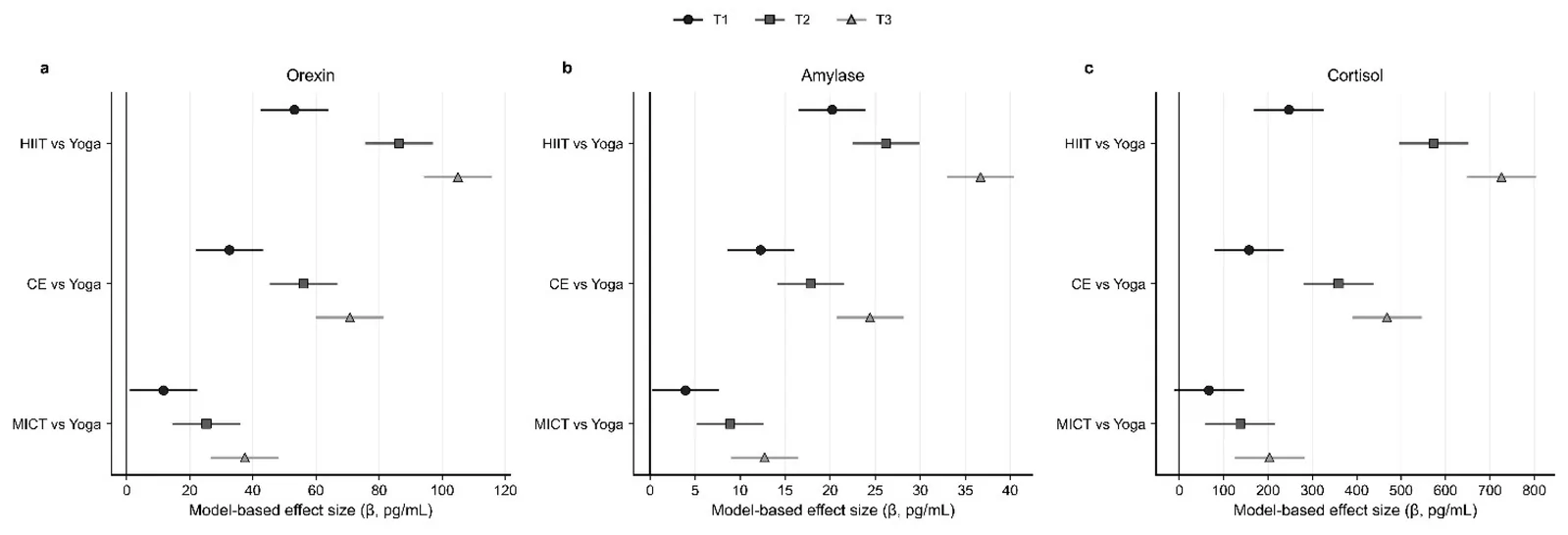
Model-based effect sizes of exercise modalities relative to control condition. Estimated effects (β coefficients ± 95% confidence intervals) derived from linear mixed-effects models comparing HIIT, CE, and MICT against the control condition (yoga) at each post-exercise time point (T1, T2, T3): (**a**) orexin; (**b**) alpha-amylase; (**c**) cortisol. All estimates are expressed relative to baseline (T0) and to the reference condition (yoga), allowing direct interpretation of exercise-induced changes over time. Across all biomarkers, effect sizes increase progressively from T1 to T3 and follow a consistent modality-dependent pattern (HIIT > CE > MICT > yoga), reflecting the different physiological demands imposed by the four exercise conditions. The vertical reference line (β = 0) indicates no difference from the control condition. Points represent model-based estimates, and horizontal lines denote 95% confidence intervals. The separation of estimates from the null line confirms statistically significant effects for most contrasts, particularly at later time points and for higher-intensity modalities. Values are expressed in the original measurement units for each biomarker (pg/mL for orexin and cortisol; U/mL for alpha-amylase), reflecting absolute differences attributable to each exercise modality.

This hierarchy was evident in both longitudinal mixed-model analyses and cumulative AUC measures, indicating a consistent pattern across analytical approaches. The magnitude and temporal dynamics of the responses suggest that exercise intensity may play an important role in acute neuroendocrine and autonomic activation, with HIIT showing the largest effects across all biomarkers (Figure 4).

## 4. Discussion

The present study compared the acute salivary responses of orexin, alpha-amylase, and cortisol following four exercise conditions with clearly different physiological demands, namely high-intensity interval training, combined exercise, moderate-intensity continuous training, and yoga, in physically active young women. The main finding was that all three biomarkers followed a consistent modality-dependent pattern, with the largest responses observed after HIIT, intermediate responses after combined exercise and MICT, and minimal variation after yoga. This hierarchy was evident both in the longitudinal mixed-model analyses and in the cumulative AUC-derived indices, supporting the robustness of the overall response pattern. Importantly, continuous heart-rate monitoring and ratings of perceived exertion confirmed that the four exercise protocols generated clearly distinct internal physiological loads, thereby strengthening the interpretation that the observed biomarker responses reflected genuine modality-dependent differences rather than inconsistencies in exercise intensity or protocol implementation. Taken together, these findings suggest that the overall physiological demands imposed by different exercise modalities are important determinants of acute neuroendocrine and autonomic activation and that a multimarker salivary approach may provide a more integrated understanding of the immediate and short-term recovery response to exercise [1,2].

Although the present findings demonstrated a clear physiological gradient across the four exercise conditions, these results should not be interpreted as reflecting exercise intensity alone. The experimental protocols differed not only in their cardiovascular demands but also in overall duration, accumulated workload, movement characteristics, and likely total energy expenditure. Consequently, the observed biomarker responses probably reflect the combined influence of multiple exercise-dose components rather than a single physiological determinant. For example, the intermittent nature of HIIT, the integrated aerobic and neuromuscular demands of combined exercise, and the continuous workload characterizing MICT may each contribute differently to autonomic and endocrine activation. Therefore, while the objective heart-rate and perceptual data confirmed that the exercise modalities elicited clearly distinct internal physiological loads, the present study was not designed to disentangle the independent contributions of exercise intensity, duration, volume, and mechanical workload [19]. Future investigations using protocols matched for total work or energy expenditure will be necessary to clarify the relative importance of these variables in shaping acute neuroendocrine responses. This interpretation is consistent with recent evidence emphasizing that the physiological response to exercise is determined by the interaction among exercise intensity, duration, total workload, and exercise modality rather than by any single component considered in isolation [20]. Consequently, the present findings should be interpreted as reflecting the integrated physiological demands imposed by each exercise modality rather than the isolated effect of exercise intensity alone.

A first relevant aspect of the present results concerns salivary orexin. Compared with cortisol and alpha-amylase, orexin should be interpreted as a less established and more exploratory peripheral biomarker. The progressive increase observed after the active exercise conditions, especially after HIIT, is compatible with the broader physiological role attributed to the orexinergic system in wakefulness, behavioral activation, energy expenditure, and autonomic regulation. However, salivary concentrations cannot be assumed to directly reflect hypothalamic release or central nervous system activity. The present findings therefore do not demonstrate central arousal activation, but rather identify a modality-dependent peripheral orexin response that may be related to the physiological demand of exercise. This pattern is of interest because direct human evidence on acute exercise-related changes in salivary orexin remains limited, particularly when orexin is examined alongside autonomic and endocrine stress markers within the same repeated-measures design [8,9].

The graded increase in salivary orexin across HIIT, combined exercise, and MICT suggests that the peripheral response may be sensitive to differences in exercise demand. HIIT likely provided the strongest overall stimulus because of its repeated vigorous efforts and rapid alternation between work and recovery. Combined exercise elicited a smaller but still marked response, potentially reflecting the integration of cardiovascular and neuromuscular demands, whereas MICT produced a more modest increase consistent with its steadier physiological profile. Yoga produced little variation over time. Nevertheless, these differences should not be interpreted as direct evidence of graded activation of central orexinergic neurons. Peripheral orexin concentrations may be influenced by secretion, transport, local production, salivary flow, assay performance, and other biological factors that were not directly assessed in the present study. Accordingly, the observed pattern should be considered hypothesis-generating and requires confirmation through studies combining salivary measurements with validated plasma, cerebrospinal, or neurophysiological indices [11].

A second key result concerns alpha-amylase. The marked increase observed after HIIT, followed by progressively smaller responses after combined exercise and MICT, strongly supports the interpretation of alpha-amylase as a sensitive marker of the immediate autonomic component of the exercise stress response. Unlike cortisol, which reflects a slower endocrine cascade, alpha-amylase appears to capture the more rapid sympathetic and adrenergic activation occurring in the early post-exercise phase [3]. In the present study, its profile was particularly informative because it clearly differentiated the exercise conditions shortly after the intervention and remained responsive across the early recovery window [1,21]. This pattern is fully consistent with the idea that exercise modalities characterized by greater cardiovascular demand, perceptual strain, and intermittent high-intensity effort produce stronger sympathetic activation than more continuous or low-intensity conditions. In practical terms, the alpha-amylase findings reinforce the usefulness of repeated salivary sampling when the aim is to detect subtle differences in autonomic load between exercise modalities [10,17].

The cortisol findings complement this interpretation but also provide a distinct temporal perspective. Cortisol showed the largest absolute changes among the three biomarkers, with a particularly pronounced increase after HIIT and, to a lesser extent, after combined exercise [22,23]. In contrast to alpha-amylase, which rose rapidly, cortisol displayed a more delayed and progressive response, especially at T2 and T3. This temporal dissociation is physiologically plausible and reflects the different regulatory pathways underlying autonomic and endocrine responses to acute exercise. While alpha-amylase is more closely related to the fast autonomic reaction to acute challenge, cortisol reflects activation of the HPA axis and the associated endocrine cascade, which unfolds more gradually over time. The present data therefore reinforce the importance of repeated post-exercise measurements, because reliance on a simple immediate post-exercise sample would likely underestimate the true magnitude of the cortisol response and would fail to capture its delayed peak. These observations are also consistent with previous studies reporting marked salivary hormonal responses following high-intensity running exercise, supporting the concept that vigorous exercise elicits a coordinated activation of autonomic and endocrine pathways. The similarity in the temporal behavior of salivary hormones across different high-intensity exercise models further supports the physiological relevance of the response pattern observed in the present study [24]. This point is methodologically important and in line with the current literature emphasizing that post-exercise biomarker kinetics are highly dependent on both exercise characteristics and recovery timing [12,25].

Considered together, the three biomarkers captured complementary temporal and physiological features of the acute exercise response. Alpha-amylase reflected the more rapid autonomic component, cortisol showed a delayed endocrine profile, and salivary orexin displayed an exploratory modality-dependent pattern [22]. Their convergence toward the same ordering of exercise conditions supports the presence of a coherent physiological-load gradient, whereas their different temporal profiles indicate that they are not interchangeable. These findings support the value of integrating markers from different physiological domains rather than relying on a single outcome.

From an applied perspective, a multimarker approach may help practitioners distinguish complementary components of the acute response to exercise. Salivary alpha-amylase may provide information on rapid autonomic activation, whereas cortisol may be more informative regarding the delayed endocrine response during early recovery. Salivary orexin, although still exploratory, may add hypothesis-generating information regarding arousal- and energy-regulation-related responses. Repeated measurements could therefore contribute to the characterization of acute training load, recovery kinetics, and individual responsiveness to different exercise modalities. However, these biomarkers should not currently be used in isolation to prescribe training or diagnose fatigue. Their practical interpretation should be integrated with external workload, heart rate, perceived exertion, sleep, recovery status, and performance measures and requires the development of reliable individual reference values before routine implementation.

The yoga condition deserves specific consideration. In the present study, yoga showed minimal variation over time for all three biomarkers and behaved as a true low-activation physiological condition. This is an important result because it confirms that not all forms of movement or structured activity induce the same acute neuroendocrine and autonomic consequences [26]. While yoga may still be beneficial in terms of relaxation, stress attenuation, or parasympathetic engagement, its acute physiological footprint appears clearly different from that of exercise modalities characterized by higher cardiovascular and metabolic demand. This interpretation is consistent with the literature indicating that even a single session of yoga or meditation-based practice may reduce stress reactivity, likely through mechanisms involving parasympathetic activation, sympathetic deactivation, and a more adaptive psychophysiological recovery profile. In our design, yoga therefore served not merely as a passive comparator, but as an active low-intensity condition with a qualitatively distinct regulatory profile. This strengthens the interpretation of the graded responses observed in the active exercise modalities [27,28].

Another strength of the present study is the choice of population. Young women remain underrepresented in exercise biomarker research, despite the importance of sex-specific physiology in stress regulation, endocrine responses, and recovery. By focusing on physically active but non-athletic women, the present study addresses a population that is ecologically relevant and scientifically important. This choice reduces the confounding influence of highly specialized training adaptations while still allowing the investigation of acute exercise responses in individuals accustomed to habitual physical activity. In this sense, the findings may be particularly useful for understanding how different exercise prescriptions influence stress-related biomarkers in an active female population outside elite sport. At the same time, the female-specific nature of the sample should be considered when interpreting the results, as direct generalization to men, sedentary individuals, or highly trained athletes should be made with caution [3,29,30].

The study also has limitations that should be acknowledged. First, the relatively small sample size (n = 15) should be acknowledged as a limitation of the present study. Nevertheless, the repeated-measures within-subject design substantially increased statistical efficiency by allowing each participant to serve as their own control, thereby reducing inter-individual variability and improving sensitivity to detect exercise-induced changes across conditions. Even so, the present findings should be interpreted with appropriate caution until they are replicated in larger and more heterogeneous cohorts. Although continuous heart-rate monitoring confirmed clear physiological separation among the four exercise modalities, exercise intensity was prescribed using age-predicted maximal heart rate rather than directly measured maximal aerobic capacity. Future studies incorporating individualized physiological thresholds, such as directly measured maximal heart rate, maximal oxygen uptake, or ventilatory thresholds, would provide an even more precise characterization of exercise intensity across different exercise modalities. Second, although the study was carefully standardized with respect to timing and sampling procedures, salivary biomarkers remain inherently sensitive to inter-individual variability and to subtle contextual influences. In addition, menstrual cycle phase and the use of hormonal contraceptives were not systematically controlled or incorporated into the analyses. These factors may influence endocrine, autonomic, and possibly orexin-related responses to exercise. Sleep duration and sleep quality before each session, as well as baseline psychological stress, were also not assessed using standardized measures. Although the repeated-measures design reduced stable between-person differences, session-specific variation in these factors may have contributed to the observed biomarker responses. Future studies should standardize or record menstrual-cycle phase, document hormonal contraceptive use, and include validated measures of sleep and perceived stress. Third, salivary orexin remains an exploratory measure, and its physiological relationship with central orexinergic signaling is not fully established. Peripheral salivary concentrations cannot be assumed to directly represent hypothalamic production, neuronal release, or central arousal. Moreover, salivary orexin measurement may be affected by pre-analytical factors, salivary flow, matrix effects, antibody specificity, cross-reactivity, and the analytical performance of the ELISA. Although samples were collected under standardized conditions and analyzed in duplicate within the same assay batch, the study did not include parallel plasma measurements, recovery experiments, dilution-linearity testing, or direct indices of central nervous system activity. The orexin findings should therefore be regarded as preliminary and hypothesis-generating rather than as direct evidence of central orexinergic activation. Finally, the current study examined only acute responses and thus cannot determine whether repeated exposure to these exercise modalities would lead to habituation, sensitization, or more stable training-related adaptations in the same biomarkers.

These limitations also point toward several directions for future research. In particular, replication studies involving larger and more heterogeneous populations are warranted to confirm the robustness and generalizability of the present findings. Such studies would also allow more detailed subgroup analyses according to sex, age, fitness level, and training status, thereby providing a broader understanding of exercise-induced neuroendocrine responses. It would also be valuable to examine whether menstrual cycle phase, sleep quality, or habitual physical activity patterns influence the acute orexin response to exercise. From a methodological perspective, future investigations could integrate salivary biomarkers with additional physiological measures, such as heart rate variability, perceptual responses, blood-based biomarkers, and neurophysiological indices, to better characterize the interplay between central arousal, autonomic regulation, and endocrine stress systems. Finally, longitudinal studies are warranted to determine whether repeated exposure to HIIT, combined exercise, MICT, or yoga leads to distinct adaptive trajectories over time in orexin, alpha-amylase, and cortisol and whether the modality-dependent response pattern observed in the present study is maintained following chronic training interventions.

## 5. Conclusions

In conclusion, acute salivary biomarker responses differed according to exercise modality in physically active young women. HIIT produced the largest changes in orexin, alpha-amylase, and cortisol, followed by combined exercise and MICT, whereas yoga produced minimal variation. The distinct temporal patterns of alpha-amylase and cortisol highlight complementary autonomic and endocrine components of the acute response. Salivary orexin also displayed a graded response, but this finding should be considered exploratory and should not be interpreted as direct evidence of central orexinergic activation. Simultaneous assessment of these biomarkers may provide a broader characterization of acute exercise load and recovery than any single marker alone. Nevertheless, the small sample, the peripheral nature of orexin measurement, and the absence of control for relevant female-specific and contextual factors require cautious interpretation and confirmation in larger, methodologically controlled studies.

## Figures and Tables

**Table 1:** Descriptive characteristics of the study participants. Values are presented as mean ± standard deviation (SD) for age, anthropometric variables (height, body mass, body mass index, and body fat percentage), and resting heart rate.

Variable	Mean ± SD
Age (years)	24.3 ± 2.8
Height (cm)	165.8 ± 5.9
Body mass (kg)	60.7 ± 7.4
Body mass index (kg/m^2^)	22.1 ± 2.3
Body fat (%)	26.8 ± 4.2
Resting heart rate (bpm)	68.5 ± 6.7

**Table 2:** Cardiovascular and perceptual indicators of internal exercise load across the four experimental exercise protocols. Values are presented as mean ± SD. Overall modality effects were evaluated using one-way repeated-measures ANOVA, and effect size is reported as partial η^2^. Pairwise comparisons were adjusted using the Holm procedure. HR = heart rate; HRmax = age-predicted maximal heart rate; HIIT = high-intensity interval training; MICT = moderate-intensity continuous training; RPE = rating of perceived exertion (Borg CR10 scale). ᵃ Absolute time spent within the prescribed target heart-rate zone is reported descriptively because the duration of the target exercise period differed across exercise modalities. *p*, probability value; *p* < 0.05 was considered statistically significant.

Variable	HIIT	Combined Exercise	MICT	Yoga	F (df)	Partial η^2^	Overall *p*
Mean heart rate (bpm)	153.2 ± 7.4	137.4 ± 6.8	132.6 ± 6.1	87.9 ± 5.8	F(3,42) = 484.46	0.972	<0.001
Peak heart rate (bpm)	181.5 ± 5.2	158.1 ± 7.2	147.6 ± 6.4	102.6 ± 6.2	F(3,42) = 485.64	0.972	<0.001
Mean % age-predicted HRmax	80.2 ± 3.8	71.9 ± 3.7	69.4 ± 3.3	46.0 ± 3.1	F(3,42) = 431.15	0.969	<0.001
Peak % age-predicted HRmax	95.0 ± 2.8	82.8 ± 3.8	77.3 ± 3.4	53.7 ± 3.3	F(3,42) = 954.41	0.986	<0.001
Time within prescribed target zone (min) ᵃ	8.6 ± 0.9	20.9 ± 2.1	32.8 ± 2.4	26.4 ± 2.0	Not applicable	Not applicable	Not applicable
Target-zone adherence (%)	86.0 ± 9.0	84.0 ± 7.0	82.0 ± 6.0	88.0 ± 6.7	F(3,42) = 3.69	0.208	0.019
Session RPE (Borg CR10)	8.4 ± 0.8	6.1 ± 0.9	4.3 ± 0.8	2.0 ± 0.6	F(3,42) = 504.68	0.973	<0.001

## Data Availability

All data are reported in the paper.
